# Identification of Parkinson's disease subtypes with distinct brain atrophy progression and its association with clinical progression

**DOI:** 10.1093/psyrad/kkae002

**Published:** 2024-02-24

**Authors:** Guoqing Pan, Yuchao Jiang, Wei Zhang, Xuejuan Zhang, Linbo Wang, Wei Cheng

**Affiliations:** School of Mathematical Sciences, Zhejiang Normal University, Jinhua 321004, China; Fudan ISTBI—ZJNU Algorithm Centre for Brain-inspired Intelligence, Zhejiang Normal University, Jinhua 321004, China; Institute of Science and Technology for Brain-Inspired Intelligence, Fudan University, Shanghai 200433, China; Key Laboratory of Computational Neuroscience and Brain-Inspired Intelligence (Fudan University), Ministry of Education, Shanghai 200433, China; Zhangjiang Fudan International Innovation Center, Shanghai 201210, China; Institute of Science and Technology for Brain-Inspired Intelligence, Fudan University, Shanghai 200433, China; Key Laboratory of Computational Neuroscience and Brain-Inspired Intelligence (Fudan University), Ministry of Education, Shanghai 200433, China; Zhangjiang Fudan International Innovation Center, Shanghai 201210, China; School of Mathematical Sciences, Zhejiang Normal University, Jinhua 321004, China; Fudan ISTBI—ZJNU Algorithm Centre for Brain-inspired Intelligence, Zhejiang Normal University, Jinhua 321004, China; Institute of Science and Technology for Brain-Inspired Intelligence, Fudan University, Shanghai 200433, China; Key Laboratory of Computational Neuroscience and Brain-Inspired Intelligence (Fudan University), Ministry of Education, Shanghai 200433, China; Zhangjiang Fudan International Innovation Center, Shanghai 201210, China; Institute of Science and Technology for Brain-Inspired Intelligence, Fudan University, Shanghai 200433, China; Key Laboratory of Computational Neuroscience and Brain-Inspired Intelligence (Fudan University), Ministry of Education, Shanghai 200433, China; Zhangjiang Fudan International Innovation Center, Shanghai 201210, China; Shanghai Medical College and Zhongshan Hospital Immunotherapy Technology Transfer Center, Shanghai 200032, China

**Keywords:** Parkinson's disease, subtypes, structural MRI, longitudinal atrophy rate

## Abstract

**Background:**

Parkinson's disease (PD) patients suffer from progressive gray matter volume (GMV) loss, but whether distinct patterns of atrophy progression exist within PD are still unclear.

**Objective:**

This study aims to identify PD subtypes with different rates of GMV loss and assess their association with clinical progression.

**Methods:**

This study included 107 PD patients (mean age: 60.06 ± 9.98 years, 70.09% male) with baseline and ≥ 3-year follow-up structural MRI scans. A linear mixed-effects model was employed to assess the rates of regional GMV loss. Hierarchical cluster analysis was conducted to explore potential subtypes based on individual rates of GMV loss. Clinical score changes were then compared across these subtypes.

**Results:**

Two PD subtypes were identified based on brain atrophy rates. Subtype 1 (n = 63) showed moderate atrophy, notably in the prefrontal and lateral temporal lobes, while Subtype 2 (n = 44) had faster atrophy across the brain, particularly in the lateral temporal region. Furthermore, subtype 2 exhibited faster deterioration in non-motor (MDS-UPDRS-Part Ⅰ, *β* = 1.26 ± 0.18, *P* = 0.016) and motor (MDS-UPDRS-Part Ⅱ, *β* = 1.34 ± 0.20, *P* = 0.017) symptoms, autonomic dysfunction (SCOPA-AUT, *β* = 1.15 ± 0.22, *P* = 0.043), memory (HVLT-Retention, *β* = −0.02 ± 0.01, *P* = 0.016) and depression (GDS, *β* = 0.26 ± 0.083, *P* = 0.019) compared to subtype 1.

**Conclusion:**

The study has identified two PD subtypes with distinct patterns of atrophy progression and clinical progression, which may have implications for developing personalized treatment strategies.

## Introduction

Parkinson's disease (PD) is the second most prevalent neurodegenerative disease, affecting an estimated 6.1 million people worldwide in 2016 alone and having a large effect on society (Feigin *et al*., 2019). PD is characterized by a range of motor and non-motor symptoms, including typical motor symptoms such as bradykinesia, rigidity, and tremor, as well as typical non-motor symptoms such as olfactory dysfunction, cognitive impairment, psychiatric symptoms, sleep disorders, and autonomic dysfunction (Kalia & Lang, 2015; Thenganatt & Jankovic, 2014; Wang *et al*., 2020). The clinical manifestations and prognosis of PD vary widely, suggesting that it may not be a singular entity (Thenganatt & Jankovic, 2014). Identifying PD subtypes is crucial for understanding the underlying pathophysiological mechanisms, stratifying patients with different progressions, and designing personalized clinical trials (Fereshtehnejad & Postuma, 2017). Although increasing evidence supports the existence of different subtypes within PD (Fereshtehnejad *et al*., 2017; Horsager *et al*., 2020; Lewis *et al*., 2005; Thenganatt & Jankovic, 2014; Zhang *et al*., 2019), current classifications are primarily based on cross-sectional data, which may mix the classification of subtypes with varying disease stages (Fereshtehnejad & Postuma, 2017; Young *et al*., 2018). To date, no studies have been reported PD subtyping based on longitudinal neuroimaging data to disentangle disease stages from disease subtypes.

The pathological mechanism of PD is the intracellular accumulation of misfolded alpha-synuclein, a presynaptic protein that forms abnormal cytoplasmic aggregates (Choi *et al*., 2022; Kalia & Lang, 2015; Lau *et al*., 2020). The misfolded alpha-synuclein can spread across distinct brain regions via the brain connectome, causing neuronal death and atrophy in related brain regions (Bloem *et al*., 2021; Henderson *et al*., 2019; Lau *et al*., 2020; Rahayel *et al*., 2022). Mouse models have demonstrated that the patterns of alpha-synuclein aggregation are closely associated with brain connectivity (Henderson *et al*., 2019). Studies in humans have also found a relationship between brain connectivity and clinical symptoms as well as atrophy progression in PD (De Micco *et al*., 2021; Vo *et al*., 2023). However, humans vary in their brain connectivity, suggesting that the alpha-synuclein aggregation pattern in PD patients also exhibits a high level of heterogeneity (Karahan *et al*., 2022; Zhang *et al*., 2017). Furthermore, other demographic characteristics, such as genetic factors and lifestyles also relate to the progression of PD (Guo *et al*., 2019; Li *et al*., 2023; Pu *et al*., 2022; Saunders-Pullman *et al*., 2018). These previous findings suggest that there may be different subtypes of PD with distinct patterns of brain atrophy.

In the present study, we aimed to identify PD subtypes with different rates of brain atrophy derived using voxel-based morphometry (VBM) with longitudinal imaging data. VBM provides an automated, quantitative analysis of gray matter distribution with high regional specificity (Ashburner & Friston, 2000). PD involves axonal degeneration and neuronal cell death, which are indexed by gray matter atrophy (Banwinkler *et al*., 2022; Yau *et al*., 2018; Zeighami *et al*., 2015). We hypothesized that the rate of gray matter volume (GMV) loss can reflect heterogeneity in PD, enabling its use for subtyping and exploration of subtype differences. We first fitted the rate of GMV loss for each patient at regional level, then explored whether these regional rates of GMV loss could classify PD patients into different subtypes. Furthermore, the observed subtypes were validated through a systematic assessment of their differences in clinical progression, cerebrospinal fluid (CSF) biomarkers, and dopamine transporter (DAT) binding deficit on single-photon emission computed tomography (SPECT) imaging.

## Materials and methods

### Participants

**Figure 1:**
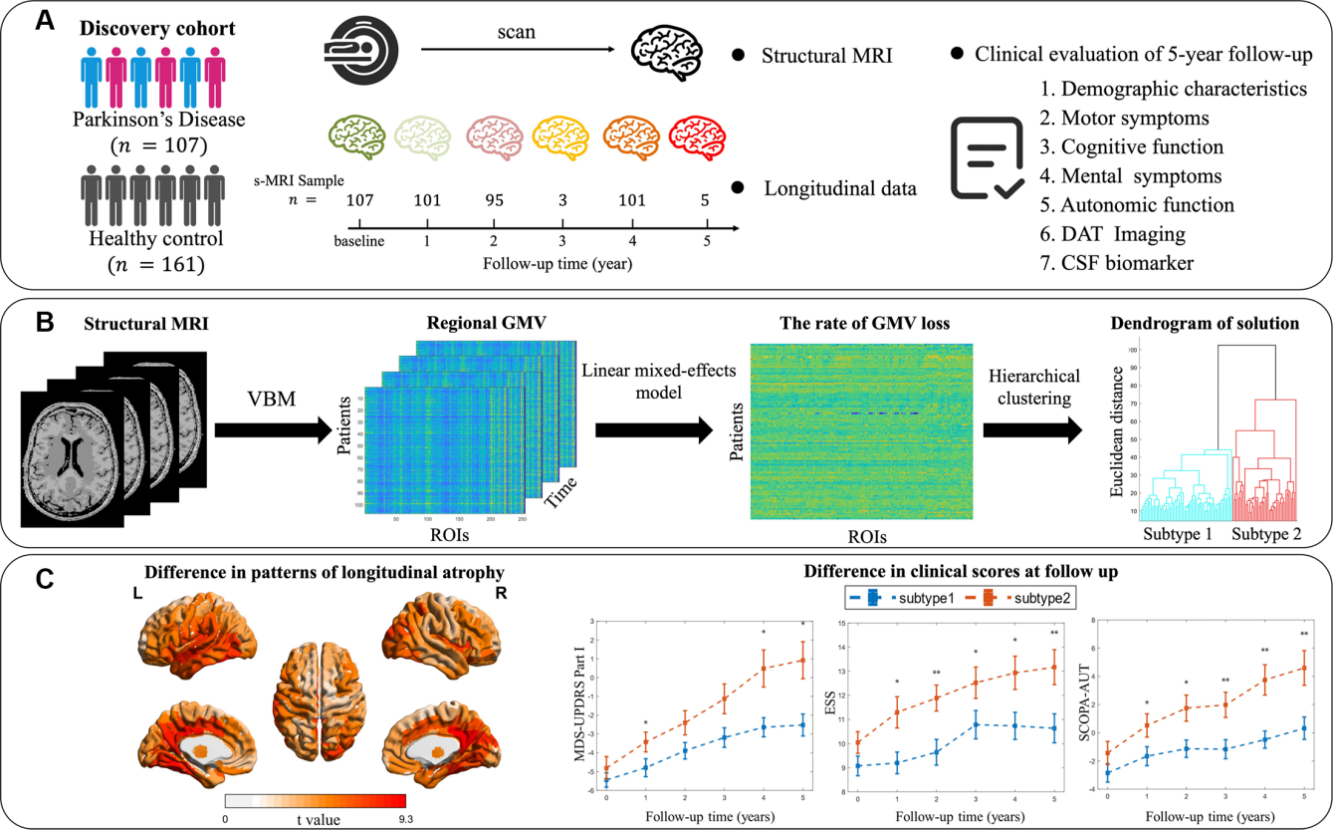
Schematic overview of the design. (**A**) A total of 268 participants with follow up structural MRI and clinical assessments were included in the study. (**B**) Hierarchical clustering was used to classify PD patients into different subtypes. First, VBM was performed on the T1-MRI scans by using the CAT 12. Then, LME was used to estimate the rate of GMV loss. Hierarchical clustering was employed to identify subtypes of patients, after normalizing the rate of GMV loss. (**C**) The differences in the rate of GMV loss and longitudinal change rate in clinical scores were compared between these subtypes to assess their differences. CAT 12 = Computational Anatomy Toolbox 12.

The Parkinson's Progression Markers Initiative (PPMI) is a landmark observational, longitudinal database consisting of neuroimaging, biological tests, and clinical and behavioral assessments (Marek *et al*., 2018). PPMI included individuals diagnosed with PD and healthy controls (HC). Recruitment criteria of patients with PD included: age ≥30, PD diagnosis within 2 years, Hoehn and Yahr Stage I–II at baseline, untreated with PD medications (levodopa, dopamine agonists, MAO-B inhibitors, or amantadine), and with one of at least two from resting tremor, bradykinesia, or rigidity (must have either resting tremor or bradykinesia) or a single asymmetric resting tremor or asymmetric bradykinesia (Marek *et al*., 2018). Furthermore, all PD patients were confirmed by the positive DAT-SPECT (Marek *et al*., 2018). To ensure robustness of the estimated rates of GMV loss, PD patients were excluded if fewer than two structural MRIs were collected or if their longest scanning interval was <36 months. HC have no current neurological disorder, no first-degree relative with PD, and normal DAT-SPECT imaging (Marek *et al*., 2018). Additionally, we excluded HC participants who were younger than 30 years old, failed image quality control, or lacked baseline data. Ultimately, a total of 161 HC and 107 PD participants were included in the study, as outlined in Supplementary Fig. S1. The overall schema of the study is presented in Fig. 1.

The data used in this study were retrieved from the PPMI database in April 2021. The PPMI study was approved by institutional review boards at each study site, and all participants provided written informed consent before enrolment.

### Clinical assessments

A comprehensive set of clinical assessments were collected in the PPMI study, in which derived variable definitions and score calculations are available. The baseline and annual follow-up clinical scores were used in our analysis. In line with previous studies (Fereshtehnejad *et al*., 2017; Pagano *et al*., 2018; Wang *et al*., 2020), we focused on clinical features that capture major PD symptoms, which included the following data:

1. Demographics: This section includes age, sex, race, disease duration, age of onset, and years of education.
2. Motor Function: We used the Movement Disorders Society-Unified Parkinson's Disease Rating Scale (MDS-UPDRS) to assess motor function. Part II focused on the motor experiences of daily living, while Part III examined motor function (Goetz *et al*., 2007).
3. Cognitive Testing: Global cognitive function was evaluated using the Montreal Cognitive Assessment (MoCA) total score, adjusted for age and education (Dalrymple-Alford *et al*., 2010). Verbal learning and memory were evaluated using the Hopkins Verbal Learning Test (HVLT), which assesses immediate recall, retention, and recognition-discrimination (Brandt *et al.*, 1998; Shapiro *et al*., 1999).
4. Autonomic Testing: Autonomic dysfunction was evaluated using the Scales for Outcomes in Parkinson's Disease–Autonomic total score (SCOPA-AUT) (Visser *et al*., 2004).
5. Sleep Problems: Sleep problems were evaluated using the REM sleep behavior disorder screening questionnaire (RBDSQ) and Epworth Sleepiness Scale (ESS) (Johns, 1991; Stiasny-Kolster *et al*., 2007).
6. Neurobehavior: Non-motor Experiences of Daily Living were assessed using MDS-UPDRS Part I (Goetz *et al*., 2007). Depression was evaluated using the Geriatric Depression Scale (GDS) (Yesavage, 1988). Trait and state anxiety were assessed using the State-Trait Anxiety Inventory (STAI) (Kendall *et al*., 1976). Impulse control disorders and related disorders were evaluated using the Questionnaire for Impulsive-Compulsive Disorders in Parkinson's Disease (QUIP) (Knight *et al*., 1983; Weintraub *et al*., 2006, 2009).
7. Olfactory Testing: Impaired olfaction was evaluated using the age/sex-adjusted University of Pennsylvania Smell Identification Test (UPSIT) (Doty *et al*., 1995).

### CSF and SPECT biomarkers

All participants conducted a lumbar puncture for the collection of CSF. Measurements of β-Amyloid_1–42_, total tau protein, and phosphorylated tau protein at Serine 181 were obtained for CSF samples at the University of Pennsylvania using the multiplex Luminex xMAP platform (Luminex Corp: Austin, TX, USA) with research-use-only Fujirebio-Innogenetics INNO-BIA AlzBio3 immunoassay kit-based reagents (Innogenetics Inc: Harvard, MA, USA) (Marek *et al*., 2018). CSF alpha-synuclein was analyzed at a central laboratory (Covance, MA, US) using a commercially available enzyme-linked immunosorbent assay kit (Locascio *et al*., 2011; Mollenhauer *et al*., 2013).

### Imaging acquisition and processing

SPECT with the DAT tracer 123I-ioflupane was obtained for most of patients at baseline, 1, 2, and 4 years of follow-up. The specific binding ratios (SBR) was calculated for all the striatal areas using the occipital lobe as a reference region (Marek *et al*., 2018).

Structural MRI scans were acquired in the sagittal plane on 3T scanners at each study site using a magnetization-prepared rapid-acquisition gradient echo sequence (Marek *et al*., 2018). Acquisition parameters were as follows: repetition time = 2300/1900 ms; echo time = 2.98/2.96/2.27/2.48/2.52 ms; inversion time = 900 ms; flip angle: 9°; 256 × 256 matrix; and 1 × 1 × 1 mm^3^ isotropic voxel.

For morphometric analysis of the imaging data, structural MRI scans were processed using the Computational Anatomy Toolbox 12 (http://www.neuro.uni-jena.de/cat/) in MATLAB. The default processing pipeline was applied, which includes bias correction of field inhomogeneities, segmentation into gray and white matter and CSF, and normalization using DARTEL. GMV estimates were extracted from the left and right hemispheres for 200 cortical and 54 subcortical regions of interest (ROI) of the Neuromorphometrics Atlas (Schaefer *et al*., 2018; Tian *et al*., 2020). Raw images with low quality (CAT12 image quality rating <70%) were excluded.

### Cluster analysis

The rate of GMV loss for each ROI was evaluated through a linear mixed-effects model (LME) (Fig. 1). Subsequently, this value was standardized using the *z*-score method at the population level. To classify PD patients into subgroups, we utilized hierarchical clustering, specifically Ward's linkage method, based on the standardized values and Euclidean distance. Hierarchical clustering offers a clear understanding of the hierarchical relationships among data points, allowing for the revelation of potential group structures without the need for *a priori* determination of the number of clusters. Euclidean distance was computed using all 254 dimensions, ensuring comprehensive consideration of the data. The Ward's linkage method, which aims to minimize variance, was used to successively merge the two closest participants, resulting in a hierarchical tree structure of larger clusters. To determine the optimal number of clusters, we employed the Calinski–Harabasz criterion (1974). This criterion evaluates the clustering effect by considering the within-cluster compactness and between-cluster separation. A larger Calinski–Harabasz index indicates a better hierarchical clustering effect, indicating that the sample is more appropriately divided into two clusters, as depicted in Supplementary Fig. S2.

To validate the clustering results, we also used *k*-means clustering on the first two principal components of atrophy rates across different brain regions. The Cohen *κ* agreement rate between hierarchical clustering and *k*-means clustering was 0.63, indicating substantial agreement between the two methods. This suggests that the patterns identified by the two different clustering techniques were consistent.

### Statistical analyses

The two-sample *t*-test was used to test the statistical difference of continuous demographic and clinical scores with the adjustment of potential confounders, including age, sex, years of education, race, and study site effect. The *χ*^2^ test was used to evaluate gender distribution. For every demographic and clinical feature, we calculated the mean and standard deviation (SD). False discovery rate (FDR) correction was applied to set the threshold for statistical significance at *P* < 0.05. All missing data points were excluded from our analyses. The covariates used in each statistical analysis are presented in Supplementary Fig. S3.

To evaluate the rate of GMV loss, we used LME using the 'fitlme' function in MATLAB software (v.R2022a, MathWorks). Age, sex, race, sites, follow-up time, group, and the interaction between time and group were included as fixed effects. Intercept and follow-up time were also included for each participant as random effects. Whenever GMV served as the outcome measure, the total intracranial volume (TIV) was adjusted. The following formulas were used for the models:

Model 1: GMV ∼ 1 + age + gender + race + years of education + site + TIV + follow-up time + (1 + follow-up time| participant)
Model 2: GMV ∼ 1 + age + gender + race + years of education + site + TIV + follow-up time × group + (1 + follow-up time | participant)
Model 3: Score ∼ 1 + age + gender + race + years of education + site + follow-up time × group + (1 + follow-up time | participant)

Model 1 was designed to fit the rate of GMV loss over time, model 2 was designed to examine the differences in GMV loss rates among different groups, and model 3 was designed to examine the differences in clinical progression among different groups. Multiple comparison corrections were made using the FDR method, and *P* values <0.05 were considered statistically significant.

## Results

### Demographic and clinical characteristics

This study included a total of 161 HC, among whom 104 (64.60%) were male, and 107 PD patients, including 75 males (70.09%). The mean age of PD patients was 60.06 ± 9.98 years, and the average disease duration at baseline was 6.97 ± 7.24 months. The mean follow-up time of structural MRI in PD patients is 4.06 years. Their mean MDS-UPDRS Part I-III scores of PD patients were 4.66 ± 3.56, 5.14 ± 3.73, and 20.63 ± 9.52, respectively. A comprehensive overview of the demographic and clinical characteristics of the PD patients and HC are displayed in Supplementary Table S1. There were significant differences in MDS-UPDRS, RBDSQ, GDS, SCOPA-AUT, STAI, and UPSIT between HC and PD at baseline.

### Two PD subtypes identified by the rate of GMV loss

**Figure 2:**
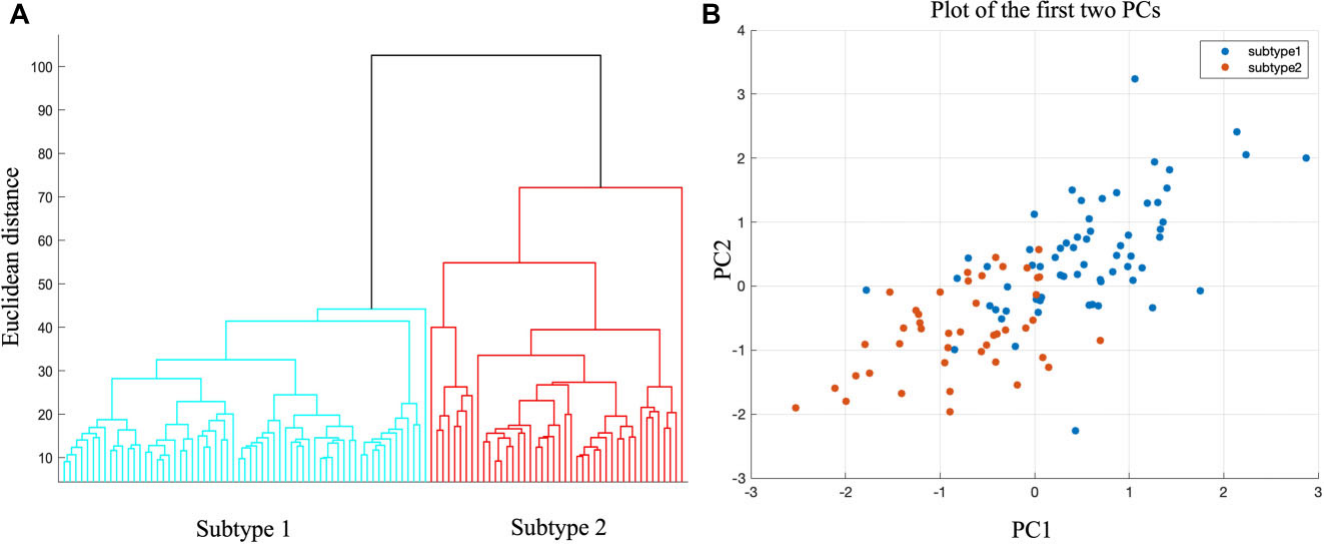
The hierarchical clustering results and its visualization. (**A**) Dendrogram of the hierarchical cluster of patients with PD. (**B**) Visual representation of the hierarchical clustering result based on the principal component (PC) coordinates of the first two dimensions.

We then investigated whether the rates of GMV loss could cluster patients into distinct groups. As shown in Fig. 2A, the results of hierarchical clustering revealed the presence of two subtypes of PD patients. To visualize the differences between these subtypes, we applied dimensionality reduction to the atrophy rates across various brain regions and plotted a scatter diagram of the first two principal components. This diagram illustrates the distinct distribution patterns observed among the two subtypes (Fig. 2B).

To assess the stability of these clusters, we conducted a leave-one-out jack-knife validation. In this analysis, we found that each validation resulted in a highly stability clustered assignment, with Dice's coefficients ranging from 0.89 ± 0.159 (mean ± standard deviation; Supplementary Fig. S4A, upper panel). Furthermore, the atrophy rate patterns within each cluster were found to be stable, with high spatial correlation coefficients compared to the main results (Subtype 1: *r* = 0.95 ± 0.149; Subtype 2: *r* = 0.99 ± 0.0052; all PFDR < 0.05; Supplementary Fig. S4B, upper panel). Additionally, we performed a 5-fold cross-validation to further test the robustness of our findings. Even with this greater perturbation to the data, each validation showed a relatively high cluster assignment, with Dice's coefficients ranging from 0.87 ± 0.124 (mean ± standard deviation; Supplementary Fig. S4A, lower panel). The atrophy rate patterns within each cluster also exhibited high spatial correlation coefficients compared to the main results (Subtype 1: *r* = 0.99 ± 0.0057; Subtype 2: *r* = 0.93 ± 0.0024; all PFDR < 0.05; Supplementary Fig. S4B, lower panel). Collectively, these results demonstrate the stability of the clusters based on the spatial distribution of atrophy rate patterns within each subtype.

These two subtypes comprised 58.88% (63 patients) and 41.12% (44 patients) of the PD patients. Subtype 1 is younger than Subtype 2 at baseline. Additionally, Subtype 1 exhibits milder severity of motor and non-motor symptoms compared to Subtype 2. Furthermore, Subtype 1 demonstrates higher DAT-SBR values in various subcortical brain regions and higher concentrations of CSF biomarkers relative to Subtype 2. However, after correction for multiple comparisons, these differences do not reach statistical significance. A summary of clinical, biological, and cognitive characteristics of the two PD subtypes is provided in Table 1.

**Table 1:** Characteristics of HC and two PD subtypes.

	HC (*n* = 161)	Subtype 1 (*n* = 63)	Subtype 2 (*n* = 44)
**Demographic features**			
Age, year, mean (SD)	60.43 (11.33)	58.79 (10.56)	61.89 (8.87)
Sex, male, *n* (%)	104 (64.6)^c^	39 (61.9)^c^	36 (81.8)*
Disease duration, mean (SD)		6.18 (6.40)	8.09 (8.26)
Years of education, mean (SD)	16.11 (2.97)	15.35 (2.96)	15.64 (2.67)
**Clinical characteristics, mean (SD)**			
MDS-UPDRS Part I	2.89 (3.06)^c^	4.03 (2.90)	5.567(4.21)^a^
MDS-UPDRS Part II	0.43 (1.00)*	4.56 (2.96)^a^	5.98 (4.52)^a^
MDS-UPDRS Part III	1.18 (2.25)*	18.95 (9.07)^a^	23.05 (9.73)^a^
MDS-UPDRS total	4.44 (4.53)*	27.54 (11.78)^a^	34.59 (15.13)^a^
RBDSQ	2.87 (2.24)^c^	3.57 (2.12)	4.86 (3.04)^a^
GDS	1.24 (2.10)	2.06 (2.29)	1.98 (1.82)
SCOPA-AUT	5.77 (3.80)^c^	7.71 (5.00)	10.28 (6.30)^a^
ESS	5.5 (3.47)	5.62 (3.11)	6.66 (3.39)
QUIP	0.26 (0.72)	0.25 (0.59)	0.25 (0.53)
STAI	56.70 (13.63)	62.48 (16.16)	65.98 (17.12)
UPSIT	33.94 (4.82)*	23.43 (7.96)^a^	17.36 (8.13)^a^
MoCA	28.26 (1.10)^c^	27.98 (1.87)	27.11 (2.42)^a^
Semantic Fluency total score	51.68 (11.18)	51.56 (10.97)	46.16 (10.09)
HVLT Immediate Recall	26.10 (4.62)	26.92 (4.71)	23.95 (5.44)
HVLT Discrimination	10.06 (2.89)	10.42 (2.15)	8.59 (4.08)
HVLT Retention	0.90 (0.19)	0.88 (0.18)	0.83 (0.20)
**DAT imaging pathology**			
Caudate	2.96 (0.64)*	2.00 (0.50) ^a^	1.85 (0.52)^a^
Putamen	2.12 (0.57) *	0.88 (0.27)^a^	0.72 (0.21)^a^
Striatum	2.54 (0.58)*	1.44 (0.37)^a^	1.28 (0.34)^a^
***CSF pathology***			
Aβ_1–42_	1005 (475)	883 (352)	814 (331)
alpha-Synuclein	1681 (758)	1519 (596)	1342 (622)
T-tau	192 (83)	169 (53)	149 (40)
P-tau	17.740 (8.88)	14.95 (5.11)	12.64 (3.69)

^a^Corrected *P* < 0.05 (versus HC); ^b^Corrected *P* < 0.05(versus subtype 1); ^c^Corrected *P* < 0.05 (versus subtype 2); *Corrected *P* < 0.05 (versus all others).

Abbreviations: Aβ_1–42_ = β-amyloid 1–42; GDS = 15-item GDS; P-tau = phosphorylated tau; T-tau = total tau.

### Comparison of rates of GMV loss between subtypes of PD and HC

**Figure 3:**
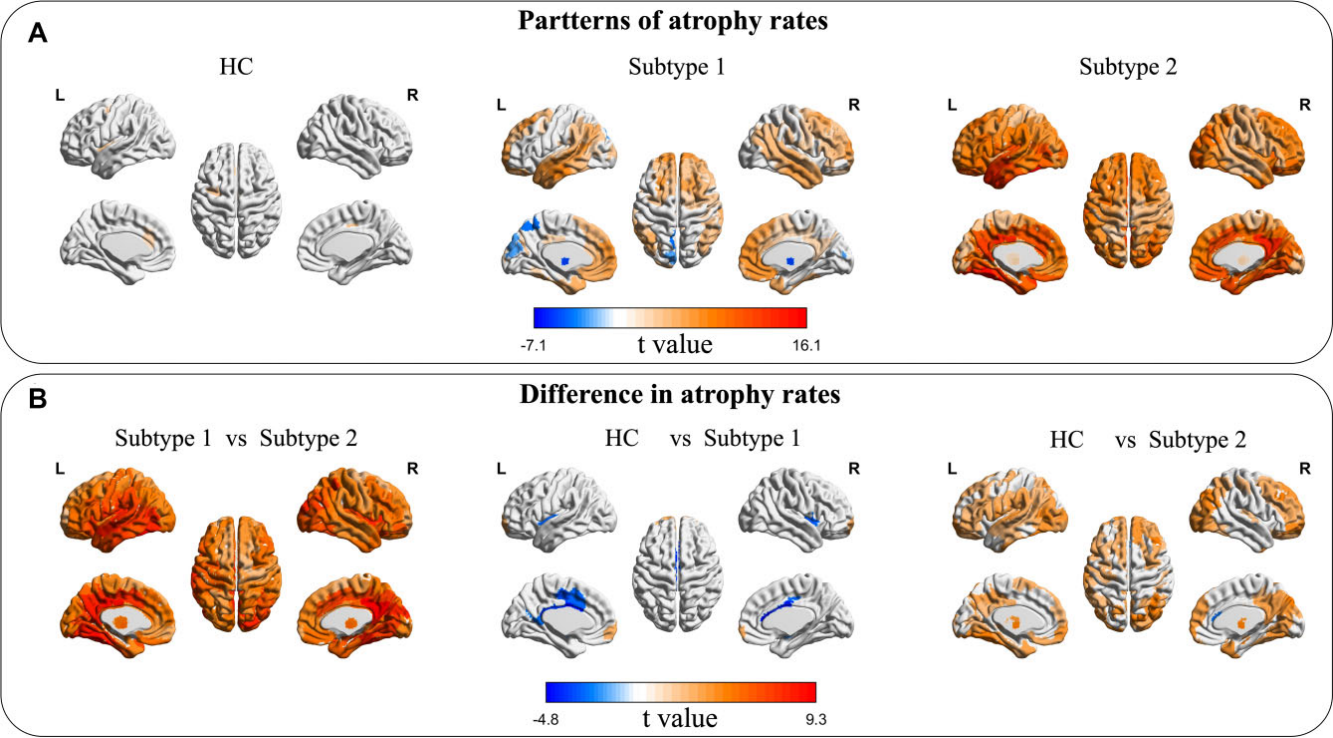
Patterns of GMV loss rates among both subtypes of PD and HC participants over time. (**A**) The patterns of GMV loss rate for each respective group. (**B**) The differences in the rate of GMV loss between each pair of groups. A higher *t* value indicates a faster rate of GMV loss or a larger difference in GMV loss rate between pairs of groups. Only ROI with statistically significant corrected *P* values, as determined through FDR correction with a threshold of *P* < 0.05, were visualized in the figures.

We further compared the rates of GMV loss between PD subtypes and HC. As shown in Fig. 3A, there were differences in the rates of GMV loss among HC and the two PD subtypes. In general, the rates of GMV loss over time was minimal within the HC group. By contrast, subtype 1 displayed a moderate level of GMV loss over time, primarily located in the prefrontal and temporal lobes. However, subtype 2 exhibited widespread higher rates of GMV loss over time in most regions of the brain. When comparing these two subtypes, subtype 2 had a significantly higher rates of GMV loss in nearly all brain regions, particularly in the lateral temporal lobe, hippocampus, and thalamus (as illustrated in Fig. 3B; *P* < 0.05, FDR corrected). These findings underscore the presence of unique and distinguishable patterns of neurodegeneration rates that are linked with the different subtypes of PD.

In addition, to gain more detailed information about the specific atrophy patterns associated with each subtype, we performed a comparative analysis of GMV data between each subtype at each follow-up visit and the corresponding values obtained from HC at baseline. Our analyses revealed that subtype 1 showed significant atrophy primarily in the superior frontal gyrus and caudate nucleus, whereas subtype 2 presented with marked atrophy in various regions including the temporal lobe, frontal lobe, and subcortical regions when compared to HC participants (refer to Supplementary Fig. S5).

### Comparison of clinical progression between two PD subtypes

**Figure 4:**
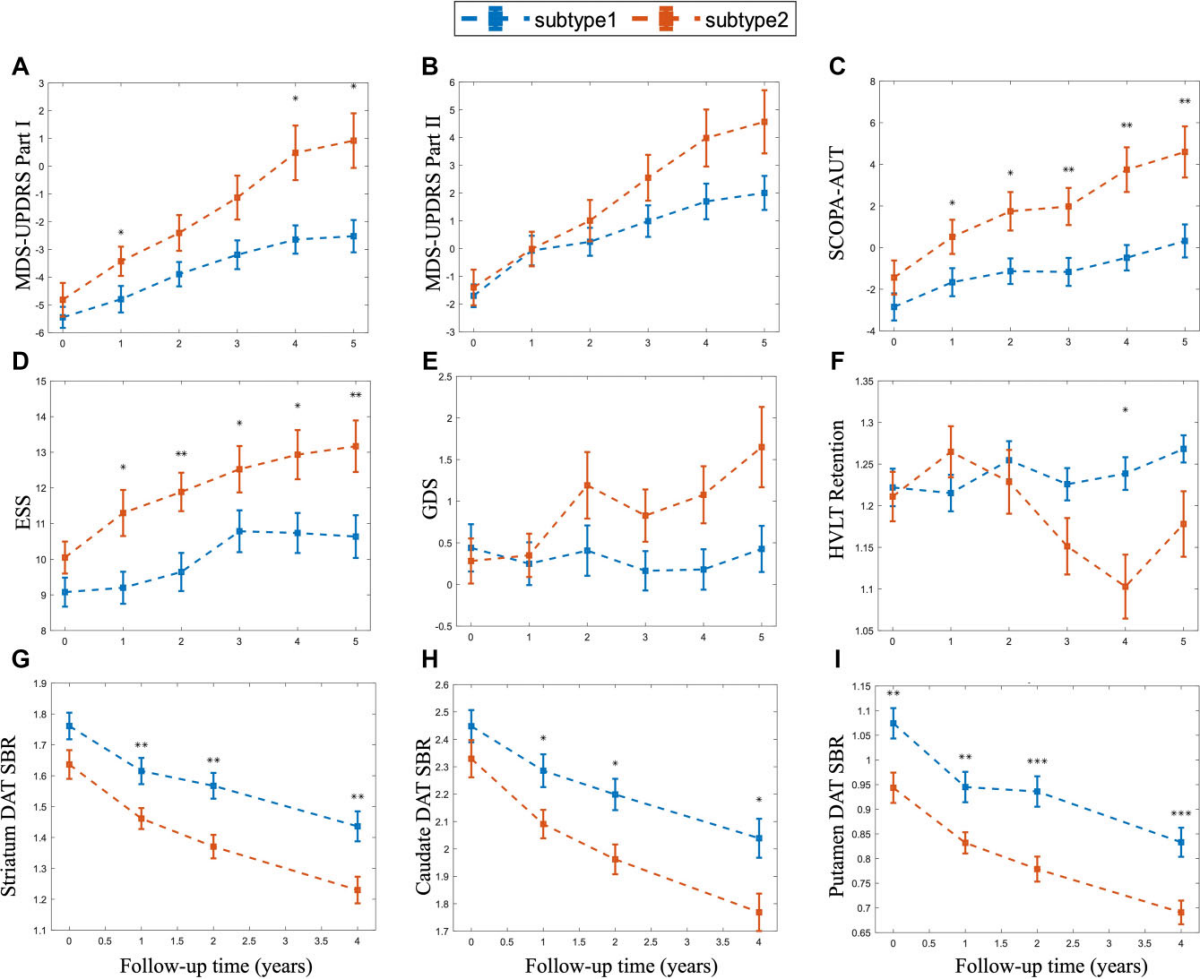
Longitudinal trajectories of clinical scores and SPECT biomarkers for two PD subtypes. The asterisks represent the statistical significance of the comparison between the two subtypes in the clinical variables at different follow-up visits. The significance levels are indicated as follows: *P < 0.05, **P < 0.01, ***P < 0.005, with the FDR correction applied for the follow-up visits. FDR correction performed in all follow-up times.

The PPMI patients were followed up for 5 years, allowing us to compare the clinical progression difference between two subtypes (Table 2). LME models were used to examine the association between PD subtypes and the rate of change in each clinical score, adjusting for covariates. Subtype 2 had a higher rate of change in MDS-UPDRS part I [group × time: *β* = 0.62; 95%CI (0.24, 1.00); *P* = 0.016], MDS-UPDRS part II [group × time: *β* = 0.61; 95%CI (0.20, 1.02); *P* = 0.017], SCOPA-AUT [group × time: *β* = 0.60; 95%CI (0.12, 1.07); *P* = 0.043], ESS [group × time: *β* = 0.23; 95%CI (−0.11, 0.56); *P* = 0.307], GDS [group × time: *β* = 0.27; 95%CI (0.08, 0.45); *P* = 0.019], and HVLT-Retention [group × time: *β* = −0.03; 95%CI (−0.05,−0.01); *P* = 0.016] compared to subtype 1 (Fig. 4A–F). Supplementary Fig. S6 demonstrates the progression disparity between the two subtypes in terms of various additional clinical scores, with no significant statistical differences observed.

**Table 2:** Longitudinal changes in clinical scores of two PD subtypes (*n* = 107).

	Subtype 1 (*n* = 63)	Subtype 2 (*n* = 44)	FDR *P* value
**Clinical characteristics**			
MDS-UPDRS Part Ⅰ	0.63 (0.10)	1.26 (0.18)	0.016
MDS-UPDRS Part Ⅱ	0.71 (0.11)	1.34 (0.20)	0.017
MDS-UPDRS Part Ⅲ	1.92 (0.32)	1.84 (0.42)	0.759
MDS-UPDRS total	3.20 (0.45)	4.10 (0.78)	0.400
RBDSQ	0.19 (0.06)	0.25 (0.07)	0.576
GDS	−0.01 (0.06)	0.26 (0.08)	0.019
SCOPA-AUT	0.56 (0.13)	1.15 (0.22)	0.043
ESS	0.39 (0.01)	0.62 (0.01)	0.307
QUIP	0.02 (0.02)	0.05 (0.03)	0.416
STAI	−0.29 (0.31)	0.68 (0.51)	0.166
MoCA	0.04 (0.05)	−0.20 (0.11)	0.073
Semantic Fluency total score	0.39 (0.22)	−0.02 (0.33	0.400
HVLT Immediate Recall	0.03 (0.10)	−0.32 (0.17)	0.116
HVLT Discrimination	0.20 (0.06)	0.27 (0.13)	0.576
HVLT Retention	0.008 (0.004)	−0.023 (0.009)	0.016

All values presented are the standardized beta coefficients (accompanied by their standard errors), unless explicitly stated otherwise.

*P* values represent the significance of the interaction term between 'follow-up time' and 'group' in model 3, with values considered significant after applying the FDR correction (*P* < 0.05).

Although there were no significant differences in DAT-SBR in the striatum, caudate, and putamen between the two subtypes at baseline and the rate of change, their difference in follow-up visits reached significant levels (*P* < 0.05 FDR; Fig. 4G–I). Subtype 1 had higher CSF levels of alpha-synuclein, t-tau, and p-tau181 than subtype 2, both at baseline and at follow-up. However, these differences in CSF biomarkers did not survival FDR correction (Fig. S7). In addition, we employed Cohen's *d* to evaluate the effect size of differences in clinical assessments for the two PD subtypes at baseline and follow-up, respectively (see Supplementary Tables S2 and S3).

## Discussion

The current study identified two PD subtypes with distinct rates of brain atrophy and clinical progression. Subtype 1 was characterized by moderate levels of atrophy rates in the prefrontal lobe and lateral temporal lobe, along with slower clinical progression. By contrast, subtype 2 exhibited higher rates of atrophy across most brain regions, as well as more severe DAT deficits. This subtype also demonstrated faster clinical progression in terms of mental, sleep, autonomic, and cognitive symptoms. The identification of distinct subtypes may aid in the development of more targeted and personalized treatment strategies for PD patients.

Our study identified two PD subtypes with different atrophy and clinical progression by using longitudinal structural MRI scans. The observed brain atrophy rates in both PD subtypes were notably heightened compared to those of the control group, thereby confirming that pathological protein aggregation indeed accelerates neuronal degeneration in PD (Choi *et al*., 2022; Lau *et al*., 2020; Shahnawaz *et al*., 2020). To proactively curb the progression of PD, future research endeavours should concentrate on devising strategies aimed at mitigating neuron loss or exploring the potential of personalized iPSC-derived dopamine progenitor cell transplantation (Schweitzer *et al*., 2020). Subtype 1 was associated with a relatively well-preserved brain structure and slower clinical progression. By contrast, subtype 2 is associated with more widespread neurodegeneration, a more severe DAT deficit, and a faster clinical deterioration. These findings align with previous research showing that PD patients with greater brain resources exhibit greater compensatory capacity, which can enhance their ability to maintain function and potentially slows the progression of the disease (Arkadir *et al*., 2014; Gregory *et al*., 2018; Nandhagopal *et al*., 2011; Wang *et al*., 2022). Moreover, our results align with previous findings that plasma neurofilament light chain (NfL) levels positively correlate with the motor and cognitive severity and progression in PD (Lin *et al*., 2019; Ye *et al*., 2021). As NfL is a biomarker for neurodegeneration, higher plasma NfL levels suggest higher levels of neurodegeneration in the brain (Khalil *et al*., 2018; Wang *et al*., 2023). Collectively, these findings support the notion that PD is a heterogeneous disorder with distinct subtypes exhibiting different patterns of neurodegeneration and clinical progression.

Subtype 2 is characterized by higher rates of atrophy in subcortical regions and the temporal lobe, suggesting higher rate of misfolded alpha-synuclein accumulation in these regions (Abdelgawad *et al*., 2023). This finding offers a potential explanation for the more rapid progression of non-motor symptoms observed in this subtype, particularly with regards to depression, sleep disorders, and cognitive impairment. The affected brain regions—which play critical roles in functions such as emotion, autonomic control, and cognition—may underlie the faster progression of these symptoms (Rolls, 2015, 2021; Rolls *et al*., 2023). Therefore, the increased atrophy observed in these areas could contribute to the more rapid progression of non-motor symptoms (Schapira *et al*., 2017; Wilson *et al*., 2019).

After correcting for multiple comparisons, no significant differences were observed in CSF biomarkers or DAT-SBRs between the two PD subtypes. This may be attributed to the small sample size, which limits the statistical power to detect significant differences. We calculated effect sizes for the differences between these two subtypes in terms of CSF biomarkers and DAT-SBRs, with all values exceeding 0.2. The effect sizes for putamen-SBR and P-tau reached moderate levels, at 0.65 and 0.50, respectively. This result aligns with previous findings that more severe dopamine neuron loss at baseline is associated with more pronounced symptoms (Liu *et al*., 2018). It is consistent with a previous finding that lower baseline CSF Aβ_1–42_ and alpha-Synuclein were associated with faster increased motor score (Irwin *et al*., 2020). We observed that subtype 1 had higher levels of all CSF biomarkers at baseline compared to subtype 2. Given that HC generally have higher CSF biomarker levels than PD patients (Irwin *et al*., 2020; Kang *et al*., 2013) and considering CSF has important role in clearing brain metabolic waste (Wichmann *et al*., 2022), this might imply that subtype 1 patients possess a relatively stronger capacity to eliminate pathological proteins (Kang *et al*., 2013). As a result, subtype 1 appears to demonstrate less accumulation of pathological proteins in the brain, leading to milder brain atrophy and slower disease progression. This finding suggests a potential connection between CSF clearance efficiency and disease progression in PD subtypes, meriting further investigation into the underlying mechanisms.

Although our discovery of two subtypes of PD was based on longitudinal neuroimaging data, baseline differences in clinical symptoms and biomarkers may aid in early identification. Specifically, while not initially statistically significant, measures such as MDS-UPDRS total scores, RBDSQ, UPSIT, SFT, HLVT, putamen-SBR, and P-tau levels exhibited moderate-to-large effect sizes (Cohen's *d* > 0.5), indicating their potential for use in baseline differentiation. Future research should investigate the utility of these markers in distinguishing between PD subtypes at baseline. Our findings align with previous studies demonstrating that PD patients with severe RBD, cognitive impairment, hyposmia, and dopaminergic deficits on DAT imaging exhibit faster clinical progression (Pagano *et al*., 2018; Schrag *et al*., 2017). These results support the notion that PD patients with these characteristics may represent a distinct subtype associated with a more rapid disease progression (Horsager *et al*., 2020).

PD remains incurable, making the need for therapies that can slow its progression increasingly urgent (Armstrong & Okun, 2020). Our findings suggest that subtype 1 exhibits slower rates of brain atrophy and disease progression compared to subtype 2. Identifying this subgroup is crucial for guiding the development of targeted therapies. Future research exploring genetic, environmental, lifestyle, immune, and metabolic differences between these subtypes may provide insights into the underlying mechanisms and modifiable factors responsible for these distinct patterns of atrophy and clinical progressions (Ortega *et al*., 2021; Pu *et al*., 2022; Zhang *et al*., 2022). Such insights could lead to the development of therapeutic strategies that effectively preserve brain structure, slow clinical progression, and improve the quality of life for PD patients. Moreover, considering the large number of modifiable risk factors associated with PD onset and cognitive decline (Belvisi *et al*., 2020; Guo *et al*., 2019; Tan *et al*., 2016), it will be important to investigate the differences in these risk factors between subtypes to identify potential targets for intervention that can slow PD progression.

One strength of this study is the use of a multicenter cohort from the PPMI database, which has comprehensive longitudinal MRI and clinical data to investigate their differences between two subtypes. However, some limitations should be considered. First, we only used a subset of participants who met the inclusion criteria, which may introduce sample bias. Second, the method used to assess the rates of GMV loss employed a linear approach. However, given that the atrophy trajectories of some brain regions may follow non-linear patterns, future studies could use more complex, non-linear models to improve the accuracy of atrophy rate assessment over an extended disease duration. This would allow for a more comprehensive estimation of atrophy rates, providing a more accurate representation of the underlying processes. Third, the mean age of onset in subtype 2 is higher than that of subtype 1 (Table 1), although the difference was not significant (*P* = 0.129). Older age at onset was associated with fast disease progression (Ferrara *et al*., 2016). Therefore, we cannot completely rule out the role of age in the progression differences between two subtypes. Finally, this study is a single-cohort study, although PPMI recruited participants from multiple sites, our subtyping results should be validated in other cohorts.

## Conclusion

In conclusion, our findings have identified two PD subtypes that exhibit distinct rates of brain atrophy and clinical progression. The identification of these subtypes offers valuable insights into the heterogeneity of the disease and may inform the development of personalized treatment strategies. Future studies should focus on exploring the neurobiological mechanisms underlying the heterogeneity of PD.

## Supplementary Material

kkae002_Supplemental_File

## Data Availability

Data used in the preparation of this study were obtained from the PPMI database (ppmi-info.org/data). For up-to-date information on the study, visit ppmi-info.org.

## References

[bib1] Abdelgawad A, Rahayel S, Zheng Y-Q et al. (2023) Predicting longitudinal brain atrophy in Parkinson's disease using a susceptible-infected-removed agent-based model. Network Neurosci. 7:906–25. 10.1162/netn_a_00296 PMC10473281 37781140

[bib2] Arkadir D, Bergman H, Fahn S (2014) Redundant dopaminergic activity may enable compensatory axonal sprouting in Parkinson disease. Neurology. 82:1093–8. 24663231 10.1212/WNL.0000000000000243

[bib3] Armstrong MJ, Okun MS (2020) Diagnosis and treatment of Parkinson disease: a review. J Am Med Assoc. 323:548–60. 10.1001/jama.2019.22360 32044947

[bib4] Ashburner J, Friston KJ (2000) Voxel-based morphometry—the methods. Neuroimage. 11:805–21. 10860804 10.1006/nimg.2000.0582

[bib5] Banwinkler M, Dzialas V, Hoenig MC et al. (2022) Gray matter volume loss in proposed brain-first and body-first Parkinson's disease subtypes. Mov Disord. 37:2066–74. 35943058 10.1002/mds.29172

[bib6] Belvisi D, Pellicciari R, Fabbrini A et al. (2020) Risk factors of Parkinson disease. Neurology, 95:e2500–8. 32943485 10.1212/WNL.0000000000010813 PMC7682833

[bib75_946_151324] Benedict RHB, Schretlen D, Groninger L et al. (1998) Hopkins Verbal Learning Test—Revised: Normative data and analysis of inter-form and test–retest reliability. Clin Neuropsychol. 12:43–55.

[bib7] Bloem BR, Okun MS, Klein C (2021) Parkinson's disease. The Lancet. 397:2284–303. 10.1016/S0140-6736(21)00218-X 33848468

[bib9] Calinski T, Harabasz J (1974) A dendrite method for cluster analysis. Commun Stat Theory Meth. 3:1–27.

[bib10] Choi ML, Chappard A, Singh BP et al. (2022) Pathological structural conversion of α-synuclein at the mitochondria induces neuronal toxicity. Nat Neurosci. 25:1134–48. 36042314 10.1038/s41593-022-01140-3 PMC9448679

[bib11] Dalrymple-Alford JC, Macaskill MR, Nakas CT et al. (2010) The MoCA: well-suited screen for cognitive impairment in Parkinson's disease. Neurology. 75:1717. 21060094 10.1212/WNL.0b013e3181fc29c9

[bib12] De Micco R, Agosta F, Basaia S et al. (2021) Functional connectomics and disease progression in drug-naïve Parkinson's disease patients. Mov Disord. 36:1603–16. 33639029 10.1002/mds.28541

[bib14] Feigin VL, Nichols E, Alam T et al. (2019) Global, regional, and national burden of neurological disorders, 1990–2016: a systematic analysis for the Global Burden of Disease Study 2016. Lancet Neurol. 18:459–80. 30879893 10.1016/S1474-4422(18)30499-X PMC6459001

[bib15] Fereshtehnejad S-M, Postuma RB (2017) Subtypes of Parkinson's disease: what do they tell us about disease progression?. Curr Neurol Neurosci Rep, 17:34. 28324303 10.1007/s11910-017-0738-x

[bib16] Fereshtehnejad S-M, Zeighami Y, Dagher A et al. (2017) Clinical criteria for subtyping Parkinson's disease: biomarkers and longitudinal progression. Brain. 140:1959–76. 28549077 10.1093/brain/awx118

[bib18] Goetz CG, Fahn S, Martinez‐Martin P et al. (2007) Movement disorder society-sponsored revision of the unified Parkinson's disease rating scale (MDS-UPDRS): process, format, and clinimetric testing plan. Mov Disord. 22:41–7. 17115387 10.1002/mds.21198

[bib19] Gregory S, Long JD, Klöppel S et al. (2018) Testing a longitudinal compensation model in premanifest Huntington's disease. Brain. 141:2156–66. 29788038 10.1093/brain/awy122 PMC6022638

[bib20] Guo Yu, Xu W, Liu F‐T et al. (2019) Modifiable risk factors for cognitive impairment in Parkinson's disease: a systematic review and meta-analysis of prospective cohort studies. Mov Disord. 34:876–83. 30869825 10.1002/mds.27665

[bib21] Henderson MX, Cornblath EJ, Darwich A et al. (2019) Spread of α-synuclein pathology through the brain connectome is modulated by selective vulnerability and predicted by network analysis. Nat Neurosci. 22:1248–57. 31346295 10.1038/s41593-019-0457-5 PMC6662627

[bib22] Horsager J, Andersen KB, Knudsen K et al. (2020) Brain-first versus body-first Parkinson's disease: a multimodal imaging case-control study. Brain. 143:3077–88. 32830221 10.1093/brain/awaa238

[bib23] Irwin DJ, Fedler J, Coffey CS et al. (2020) Evolution of Alzheimer's disease cerebrospinal fluid biomarkers in early Parkinson's disease. Ann Neurol. 88:574–87. 32542885 10.1002/ana.25811 PMC7497251

[bib24] Johns MW (1991) A new method for measuring daytime sleepiness: the Epworth Sleepiness Scale. Sleep. 14:540–5. 1798888 10.1093/sleep/14.6.540

[bib25] Kalia LV, Lang AE (2015) Parkinson's disease. Lancet. 386:896–912. 25904081 10.1016/S0140-6736(14)61393-3

[bib26] Kang Ju-H (2013) Association of cerebrospinal fluid β-amyloid 1-42, t-tau, p-tau 181, and α-synuclein levels with clinical features of drug-naive patients with early parkinson disease. JAMA Neurol. 70:1277–87. 23979011 10.1001/jamaneurol.2013.3861 PMC4034348

[bib27] Karahan E, Tait L, Si R et al. (2022) The interindividual variability of multimodal brain connectivity maintains spatial heterogeneity and relates to tissue microstructure. Commun Biol. 5:1007. 36151363 10.1038/s42003-022-03974-w PMC9508245

[bib28] Kendall PC, Finch AJ, Auerbach SM et al. (1976) The State-Trait Anxiety Inventory: a systematic evaluation. J Consult Clin Psych. 44:406–12. 10.1037//0022-006x.44.3.406 932270

[bib29] Khalil M, Teunissen CE, Otto M et al. (2018) Neurofilaments as biomarkers in neurological disorders. Nat Rev Neurol. 14:577–89. 30171200 10.1038/s41582-018-0058-z

[bib30] Knight RG, Waal‐Manning HJ, Spears GF. (1983) Some norms and reliability data for the State-Trait Anxiety Inventory and the Zung Self-Rating Depression scale. Br J Clin Psychol. 22:245–9. 6640176 10.1111/j.2044-8260.1983.tb00610.x

[bib31] Lau A, So RWL, Lau HHC et al. (2020) α-Synuclein strains target distinct brain regions and cell types. Nat Neurosci. 23:21–31. 31792467 10.1038/s41593-019-0541-x PMC6930851

[bib32] Lewis SJG (2005) Heterogeneity of Parkinson's disease in the early clinical stages using a data driven approach. J Neurol Neurosurg Psychiatr. 76:343–8. 10.1136/jnnp.2003.033530 PMC1739569 15716523

[bib33] Li G, Huang P, Cui S et al. (2023) Effect of long-term Tai Chi training on Parkinson's disease: a 3.5-year follow-up cohort study. J Neurol Neurosurg Psychiat. 95:222–8. 10.1136/jnnp-2022-330967 37875337

[bib34] Lin C-H, Li C-H, Yang K-C et al. (2019) Blood NfL: a biomarker for disease severity and progression in Parkinson disease. Neurology. 93:e1104–11. 31420461 10.1212/WNL.0000000000008088

[bib35] Liu F-T, Ge J-J, Wu J-J et al. (2018) Clinical, dopaminergic, and metabolic correlations in Parkinson disease: a dual-tracer PET atudy. Clin Nucl Med. 43:562–71. 29863572 10.1097/RLU.0000000000002148

[bib36] Locascio JJ, Schulz-Schaeff W, Mollenhauer B et al. (2011) α-Synuclein and tau concentrations in cerebrospinal fluid of patients presenting with parkinsonism: a cohort study. Lancet Neurol. 10:297. 10.1016/S1474-4422(11)70014-X 21317042

[bib37] Marek K, Chowdhury S, Siderowf A et al. (2018) The Parkinson's progression markers initiative (PPMI) – establishing a PD biomarker cohort. Ann Clin Transl Neurology. 5:1460–77. 10.1002/acn3.644 PMC6292383 30564614

[bib38] Mollenhauer B, Trautmann E, Taylor P et al. (2013) Total CSF α-synuclein is lower in de novo Parkinson patients than in healthy subjects. Neurosci Lett. 532:44–8. 23149132 10.1016/j.neulet.2012.11.004

[bib39] Nandhagopal R, Kuramoto L, Schulzer M et al. (2011) Longitudinal evolution of compensatory changes in striatal dopamine processing in Parkinson's disease. Brain. 134:3290–8. 22075521 10.1093/brain/awr233

[bib40] Ortega RA, Wang C, Raymond D et al. (2021) Association of dual LRRK2 G2019S and GBA variations with Parkinson disease Progression. JAMA Network Open. 4:e215845. 33881531 10.1001/jamanetworkopen.2021.5845 PMC8060834

[bib17] Pagano G, Ferrara N, Brooks DJ et al. (2016) Age at onset and Parkinson disease phenotype. Neurology. 86:1400–7. 26865518 10.1212/WNL.0000000000002461 PMC4831034

[bib41] Pagano G, De Micco R, Yousaf T et al. (2018) REM behavior disorder predicts motor progression and cognitive decline in Parkinson disease. Neurology. 91:e894–905. 30089615 10.1212/WNL.0000000000006134

[bib42] Pu J‐Li, Jin C‐Y, Wang Z‐X et al. (2022) Apolipoprotein E genotype contributes to motor progression in Parkinson's disease. Mov Disord. 37:196–200. 34612548 10.1002/mds.28805

[bib43] Rahayel S, Mišić B, Zheng Y-Q et al. (2022) Differentially targeted seeding reveals unique pathological alpha-synuclein propagation patterns. Brain. 145:1743–56. 34910119 10.1093/brain/awab440 PMC9166565

[bib13] Richard L, Steven M, Matthew B (1995) Olfactory Testing as an Aid in the Diagnosis of Parkinson's Disease: Development of Optimal Discrimination Criteria. Neurodegeneration. 4:93–7. 7600189 10.1006/neur.1995.0011

[bib44] Rolls ET (2015) Limbic systems for emotion and for memory, but no single limbic system. Cortex. 62:119–57. 24439664 10.1016/j.cortex.2013.12.005

[bib45] Rolls ET (2021) The neuroscience of emotional disorders. In: Heilman K.M., Nadeau S.E. (eds). Disorders of Emotion in Neurologic Disease, 183. Elsevier, 1–26. 10.1016/B978-0-12-822290-4.00002-5 34389113

[bib46] Rolls ET, Deco G, Huang C-C et al. (2023) Human amygdala compared to orbitofrontal cortex connectivity, and emotion. Prog Neurobiol. 220:102385. 36442728 10.1016/j.pneurobio.2022.102385

[bib47] Saunders-Pullman R, Mirelman A, Alcalay RN et al. (2018) Progression in the LRRK2-asssociated Parkinson disease population. JAMA Neurol. 75:312–9. 29309488 10.1001/jamaneurol.2017.4019 PMC5885854

[bib48] Schaefer A, Kong Ru, Gordon EM et al. (2018) Local-global parcellation of the human cerebral cortex from intrinsic functional connectivity MRI. Cereb Cortex, 28:3095–114. 28981612 10.1093/cercor/bhx179 PMC6095216

[bib49] Schapira AHV, Chaudhuri KR, Jenner P (2017) Non-motor features of Parkinson disease. Nat Rev Neurosci. 18:435–50. 28592904 10.1038/nrn.2017.62

[bib50] Schrag A, Siddiqui UF, Anastasiou Z et al. (2017) Clinical variables and biomarkers in prediction of cognitive impairment in patients with newly diagnosed Parkinson's disease: a cohort study. Lancet Neurol. 16:66. 27866858 10.1016/S1474-4422(16)30328-3 PMC5377592

[bib51] Schweitzer JS, Song B, Herrington TM et al. (2020) Personalized iPSC-derived dopamine progenitor cells for Parkinson's disease. N Engl J Med. 382:1926–32. 32402162 10.1056/NEJMoa1915872 PMC7288982

[bib52] Shahnawaz M, Mukherjee A, Pritzkow S et al. (2020) Discriminating α-synuclein strains in Parkinson's disease and multiple system atrophy. Nature. 578:273–7. 32025029 10.1038/s41586-020-1984-7 PMC7066875

[bib53] Shapiro AM, Benedict RHB, Schretlen D et al. (1999) Construct and concurrent validity of the Hopkins Verbal Learning Test – Revised. Clin Neuropsychol. 13:348–58. 10726605 10.1076/clin.13.3.348.1749

[bib54] Stiasny‐Kolster K, Mayer G, Schäfer S et al. (2007) The REM sleep behavior disorder screening questionnaire – a new diagnostic instrument. Mov Disord. 22:2386–93. 17894337 10.1002/mds.21740

[bib55] Tan LC, Methawasin K, Tan E-K et al. (2016) Dietary cholesterol, fats and risk of Parkinson's disease in the Singapore Chinese health study. J Neurol Neurosurg Psychiat. 87:86–92. 25669745 10.1136/jnnp-2014-310065 PMC4929981

[bib56] Thenganatt MA, Jankovic J (2014) Parkinson disease subtypes. JAMA Neurol. 71:499–504. 24514863 10.1001/jamaneurol.2013.6233

[bib57] Tian Ye, Margulies DS, Breakspear M et al. (2020) Topographic organization of the human subcortex unveiled with functional connectivity gradients. Nat Neurosci. 23:1421–32. 32989295 10.1038/s41593-020-00711-6

[bib58] Visser M, Marinus J, Stiggelbout AM et al. (2004) Assessment of autonomic dysfunction in Parkinson's disease: the SCOPA-AUT. Mov Disord. 19:1306–12. 15390007 10.1002/mds.20153

[bib59] Vo A, Tremblay C, Rahayel S et al. (2023) Network connectivity and local transcriptomic vulnerability underpin cortical atrophy progression in Parkinson's disease. NeuroImage Clin. 40:103523. 38016407 10.1016/j.nicl.2023.103523 PMC10687705

[bib60] Wang L, Cheng W, Rolls ET et al. (2020) Association of specific biotypes in patients with Parkinson disease and disease progression. Neurology. 95:E1445–60. 32817178 10.1212/WNL.0000000000010498 PMC7116258

[bib61] Wang L, Wu P, Brown P et al. (2022) Association of structural measurements of brain reserve with motor progression in patients with Parkinson disease. Neurology. 99:e977–88. 35667838 10.1212/WNL.0000000000200814 PMC7613818

[bib62] Wang L, Zhou C, Zhang W et al. (2023) Association of cortical and subcortical microstructure with clinical progression and fluid biomarkers in patients with Parkinson disease. Neurology. 101:e300–10. 37202161 10.1212/WNL.0000000000207408 PMC10382272

[bib63] Weintraub D, Hoops S, Shea JA et al. (2009) Validation of the questionnaire for impulsive-compulsive disorders in Parkinson's disease. Mov Disord. 24:1461–7. 19452562 10.1002/mds.22571 PMC2848971

[bib64] Weintraub D, Oehlberg KA, Katz IR et al. (2006) Test characteristics of the 15-Item Geriatric Depression Scale and Hamilton Depression Rating Scale in Parkinson Disease. Am J Geriatr Psychiatry. 14:169–75. 16473982 10.1097/01.JGP.0000192488.66049.4b PMC1571046

[bib65] Wichmann TO, Damkier HH, Pedersen M (2022) A brief overview of the cerebrospinal fluid system and its implications for brain and spinal cord diseases. frontiers in human neuroscience. Front Human Neurosci. 15:737217. 10.3389/fnhum.2021.737217 PMC8813779 35126070

[bib66] Wilson H, Niccolini F, Pellicano C et al. (2019) Cortical thinning across Parkinson's disease stages and clinical correlates. J Neurol Sci. 398:31–8. 30682518 10.1016/j.jns.2019.01.020

[bib67] Yau Y, Zeighami Y, Baker TE et al. (2018) Network connectivity determines cortical thinning in early Parkinson's disease progression. Nat Commun. 9:12. 29295991 10.1038/s41467-017-02416-0 PMC5750227

[bib68] Ye R, Locascio JJ, Goodheart AE et al. (2021) Serum NFL levels predict progression of motor impairment and reduction in putamen dopamine transporter binding ratios in de novo Parkinson's disease: an 8-year longitudinal study. Parkinsonism Relat Disord. 85:11–6. 33639572 10.1016/j.parkreldis.2021.02.008 PMC8714021

[bib69] Yesavage JA (1988) Geriatric Depression Scale. Psychopharmacol Bull. 24:709–11. 3249773

[bib70] Young AL, Marinescu RV, Oxtoby NP et al. (2018) Uncovering the heterogeneity and temporal complexity of neurodegenerative diseases with subtype and stage inference. Nat Commun. 9:4273. 30323170 10.1038/s41467-018-05892-0 PMC6189176

[bib71] Zeighami Y, Ulla M, Iturria-Medina Y et al. (2015) Network structure of brain atrophy in de novo Parkinson's disease. eLife. 4:e08440. 26344547 10.7554/eLife.08440 PMC4596689

[bib73] Zhang X, Molsberry SA, Schwarzschild MA et al. (2022) Association of diet and physical activity with all-cause mortality among adults with Parkinson disease. JAMA Net Open. 5:e2227738. 10.1001/jamanetworkopen.2022.27738 PMC9391952 35984656

[bib72] Zhang Xi, Chou J, Liang J et al. (2019) Data-driven subtyping of Parkinson's disease using longitudinal clinical records: a cohort study. Sci Rep. 9:797. 30692568 10.1038/s41598-018-37545-z PMC6349906

[bib74] Zhang Y, Michel-Herve Larcher K, Misic B et al. (2017) Anatomical and functional organization of the human substantia nigra and its connections. eLife. 6:1–23. 10.7554/eLife.26653 PMC5606848 28826495

