# Supplementary material for: Identification of Parkinson's disease subtypes with distinct brain atrophy progression and its association with clinical progression
Source: Psychoradiology. 2024 Feb 24;4:kkae002. doi: 10.1093/psyrad/kkae002 (PMC10953620; doi:10.1093/psyrad/kkae002)
Supplement: kkae002_Supplemental_File [file kkae002_supplemental_file.docx]

**Identification of Parkinson's Disease Subtypes with Distinct Brain Atrophy Progression and its Association with Clinical Progression**

**Supplementary Materials**

[Supplementary Table 1. Characteristics of healthy controls and patients with PD 2](#_Toc156932860)

[Supplementary Table 2. Effect size of the difference among the two subtypes and HC at baseline 3](#_Toc156932861)

[Supplementary Table 3. Effect size of the difference between the two subtypes during follow-up 5](#_Toc156932862)

[Supplementary Figure 1: Sample selection process and its overall information in five years of follow up. 6](#_Toc156932863)

[Supplemenatry Figure 2: A graph representing the Calinski-Harabasz index for each potential number of clusters. 7](#_Toc156932864)

[Supplementary Figure 3: Covariates utilized in the statistical analyses. 8](#_Toc156932865)

[Supplementary Figure 4: Stability of cluster assignments and clustered atrophy rate-patterns. 9](#_Toc156932866)

[Supplementary Figure 6: Longitudinal trajectories of clinical scores for two PD subtypes. 11](#_Toc156932867)

[Supplementary Figure 7: Longitudinal trajectories of CSF biomarkers for two PD subtypes. 12](#_Toc156932868)

# Supplementary Table 1. Characteristics of healthy controls and patients with PD

| **Table S1 Characteristics of healthy controls and patients with PD** | | | |
| --- | --- | --- | --- |
|  | **HC (n = 161)** | **PD (n = 107)** | **P Value ^a^** |
| **Demographic features** | | | |
| *Age, year, mean (SD)* | 60.43 (11.33) | 60.06 (9.98) | 0.8958 |
| *Sex, male, n (%)* | 104 (64.6) | 75 (70.1) | > 0.1 |
| *Disease duration, mean (SD)* | NA | 6.97 (7.24) | NA |
| *Years of education, mean (SD)* | 16.12 (2.97) | 15.47 (2.84) | 0.1626 |
| **Clinical characteristics, mean (SD)** | | | |
| *MDS-UPDRS Part I* | 2.89 (3.06) | 4.66 (3.56) | 0.0040 ^b^ |
| *MDS-UPDRS Part II* | 0.43 (1.00) | 5.14 (3.73) | <0.0001^b^ |
| *MDS-UPDRS Part III* | 1.17 (2.25) | 20.64 (9.52) | <0.0001^b^ |
| *MDS-UPDRS total* | 4.44 (4.53) | 30.44 (13.65) | <0.0001^b^ |
| *RBDSQ* | 2.87 (2.24) | 4.10 (2.61) | 0.0141^b^ |
| *GDS* | 1.24 (2.10) | 2.03 (2.10) | 0.3646 |
| SCOPA-AUT | 5.77 (3.80) | 8.75 (5.69) | <0.0105^b^ |
| *ESS* | 5.50 (3.47) | 6.05 (3.25) | 0.7909 |
| *QUIP* | 0.26 (0.72) | 0.25 (0.57) | 0.4211 |
| *STAI* | 56.70 (13.63) | 63.92 (16.58) | 0.1554 |
| *UPSIT* | 33.94 (4.81) | 20.93 (8.54) | <0.0001^b^ |
| *MoCA* | 28.27 (1.11) | 27.63 (2.15) | 0.1315 |
| *Semantic fluency total score* | 51.68 (11.18) | 49.34 (10.90) | 0.3406 |
| *HVLT Immediate Recall* | 26.10 (4.62) | 25.70 (5.21) | 0.8089 |
| *HVLT Discrimination* | 10.06 (2.89) | 9.66 (3.21) | 0.4211 |
| *HVLT Retention* | 0.90 (0.19) | 0.86 (0.19) | 0.3002 |
| ^a^ p values are FDR-corrected for multiple comparisons. ^b^ Significant.  Abbreviations: ESS = Epworth Sleepiness Scale; GDS = 15-item Geriatric Depression Scale; HVLT = Hopkins Verbal Learning Test; MDS-UPDRS = Movement Disorder Society–sponsored revision of the Unified Parkinson’s Disease Rating Scale; MoCA = Montreal Cognitive Assessment; PD = Parkinson disease; QUIP = Questionnaire for Impulsive-Compulsive Disorders in Parkinson’s Disease; RBD = REM sleep behavior disorder; RBDSQ = REM Sleep Behavior Disorder Screening Questionnaire; SCOPA-AUT = Scales for Outcomes in Parkinson’s Disease–Autonomic; STAI = State-Trait Anxiety Inventory; UPSIT = University of Pennsylvania Smell Identification Test. | | | |

# Supplementary Table 2. Effect size of the difference among the two subtypes and HC at baseline

| **Table S2 Effect size of the difference among the two subtypes and HC at baseline** | | | |
| --- | --- | --- | --- |
|  | **Subtype 1 VS HC** | **Subtype 2 VS HC** | **Subtype 2 VS Subtype 1** |
| ***Demographic features*** | | | |
| *Age, year, mean (SD)* | -0.148 | 0.134 | 0.313 |
| *Sex, male, n (%)* | 0.056 | -0.373 | -0.441 |
| *Disease duration, mean (SD)* | NA | NA | 0.265 |
| *Years of education, mean (SD)* | -0.259 | -0.165 | 0.101 |
| ***Clinical characteristics, mean (SD)*** | | | |
| *MDS-UPDRS Part I* | 0.377 | 0.802 | 0.440 |
| *MDS-UPDRS Part II* | 2.315 | 2.450 | 0.386 |
| *MDS-UPDRS Part III* | 3.445 | 4.460 | 0.438 |
| *MDS-UPDRS total* | 3.155 | 3.748 | 0.532 |
| *RBDSQ* | 0.318 | 0.819 | 0.508 |
| *GDS* | 0.384 | 0.362 | -0.041 |
| *SCOPA-AUT* | 0.467 | 1.014 | 0.460 |
| *ESS* | 0.035 | 0.336 | 0.322 |
| *QUIP* | -0.010 | -0.016 | -0.007 |
| *STAI* | 0.401 | 0.642 | 0.211 |
| *UPSIT* | -1.792 | -2.917 | -0.755 |
| *MoCA* | -0.208 | -0.776 | -0.412 |
| *Semantic fluency total score* | -0.011 | -0.504 | -0.508 |
| *HVLT Immediate Recall* | 0.177 | -0.446 | -0.591 |
| *HVLT Discrimination* | 0.132 | -0.462 | -0.592 |
| *HVLT Retention* | -0.135 | -0.378 | -0.248 |
| ***DAT Imaging pathology*** | | | |
| *Caudate* | -1.589 | -1.803 | -0.299 |
| *Putamen* | -2.447 | -2.708 | -0.651 |
| *Striatum* | -2.062 | -2.313 | -0.436 |
| ***CSF pathology*** | | | |
| *Aβ _1-42_* | -0.274 | -0.426 | -0.202 |
| *α-Synuclein* | -0.226 | -0.464 | -0.292 |
| *T-tau* | -0.302 | -0.570 | -0.425 |
| *P-tau* | -0.348 | -0.633 | -0.505 |
| Abbreviations: Aβ1-42 = β-amyloid 1-42; ESS = Epworth Sleepiness Scale; GDS = 15-item Geriatric Depression Scale; HVLT = Hopkins Verbal Learning Test; MDS-UPDRS = Movement Disorder Society–sponsored revision of the Unified Parkinson’s Disease Rating Scale; MoCA = Montreal Cognitive Assessment; PD = Parkinson disease; P-tau = phosphorylated tau; QUIP = Questionnaire for Impulsive-Compulsive Disorders in Parkinson’s Disease; RBD = REM sleep behaviour disorder; RBDSQ = REM Sleep Behaviour Disorder Screening Questionnaire; SCOPA-AUT = Scales for Outcomes in Parkinson’s Disease–Autonomic; STAI = State-Trait Anxiety Inventory; T-tau = total tau; UPSIT = University of Pennsylvania Smell Identification Test. | | | |

# Supplementary Table 3. Effect size of the difference between the two subtypes during follow-up

| **Table S3 Effect size of the difference between the two subtypes during follow-up** | | | | | | |
| --- | --- | --- | --- | --- | --- | --- |
| **Follow-up year** | **Baseline** | **1-year** | **2-year** | **3-year** | **4-year** | **5-year** |
| ***Clinical characteristics, mean (SD)*** | | | | | | |
| *MDS-UPDRS Part I* | 0.440 | 0.600 | 0.559 | 0.614 | 0.724 | 0.775 |
| *MDS-UPDRS Part II* | 0.386 | 0.256 | 0.380 | 0.512 | 0.552 | 0.594 |
| *MDS-UPDRS Part III* | 0.438 | 0.124 | 0.324 | 0.420 | 0.353 | 0.262 |
| *MDS-UPDRS total* | 0.532 | 0.294 | 0.485 | 0.509 | 0.460 | 0.456 |
| *RBDSQ* | 0.508 | 0.435 | 0.577 | 0.520 | 0.520 | 0.404 |
| *GDS* | -0.041 | 0.110 | 0.326 | 0.354 | 0.455 | 0.484 |
| *SCOPA-AUT* | 0.460 | 0.591 | 0.676 | 0.738 | 0.856 | 0.740 |
| *ESS* | 0.322 | 0.515 | 0.574 | 0.373 | 0.463 | 0.511 |
| *QUIP* | -0.007 | 0.050 | -0.025 | 0.168 | 0.084 | 0.248 |
| *STAI* | 0.211 | 0.444 | 0.214 | 0.429 | 0.451 | 0.479 |
| *MoCA* | -0.412 | -0.535 | -0.587 | -0.543 | -0.685 | -0.629 |
| *Semantic fluency total score* | -0.508 | -0.396 | -0.472 | -0.460 | -0.446 | -0.534 |
| *HVLT Immediate Recall* | -0.591 | -0.526 | -0.262 | -0.552 | -0.816 | -0.608 |
| *HVLT Discrimination* | -0.592 | -0.425 | -0.451 | -0.329 | -0.606 | -0.868 |
| *HVLT Retention* | -0.248 | 0.085 | -0.257 | -0.545 | -0.730 | -0.616 |
| ***DAT Imaging pathology*** | | | | | | |
| *Caudate* | -0.299 | -0.506 | -0.550 | NA | -0.563 | NA |
| *Putamen* | -0.651 | -0.635 | -0.796 | NA | -0.778 | NA |
| *Striatum* | -0.436 | -0.589 | -0.660 | NA | -0.655 | NA |
| ***CSF pathology*** | | | | | | |
| *Aβ_1-42_* | -0.202 | -0.108 | -0.107 | -0.035 | NA | NA |
| *α-Synuclein* | -0.292 | -0.413 | -0.394 | -0.498 | NA | NA |
| *T-tau* | -0.425 | -0.439 | -0.373 | -0.461 | NA | NA |
| *P-tau* | -0.505 | -0.440 | -0.300 | -0.378 | NA | NA |
| Abbreviations: Aβ_1-42_ = β-amyloid 1-42; ESS = Epworth Sleepiness Scale; GDS = 15-item Geriatric Depression Scale; HVLT = Hopkins Verbal Learning Test; MDS-UPDRS = Movement Disorder Society–sponsored revision of the Unified Parkinson’s Disease Rating Scale; MoCA = Montreal Cognitive Assessment; PD = Parkinson disease; P-tau = phosphorylated tau; QUIP = Questionnaire for Impulsive-Compulsive Disorders in Parkinson’s Disease; RBD = REM sleep behaviour disorder; RBDSQ = REM Sleep Behaviour Disorder Screening Questionnaire; SCOPA-AUT = Scales for Outcomes in Parkinson’s Disease–Autonomic; STAI = State-Trait Anxiety Inventory; T-tau = total tau. | | | | | | |


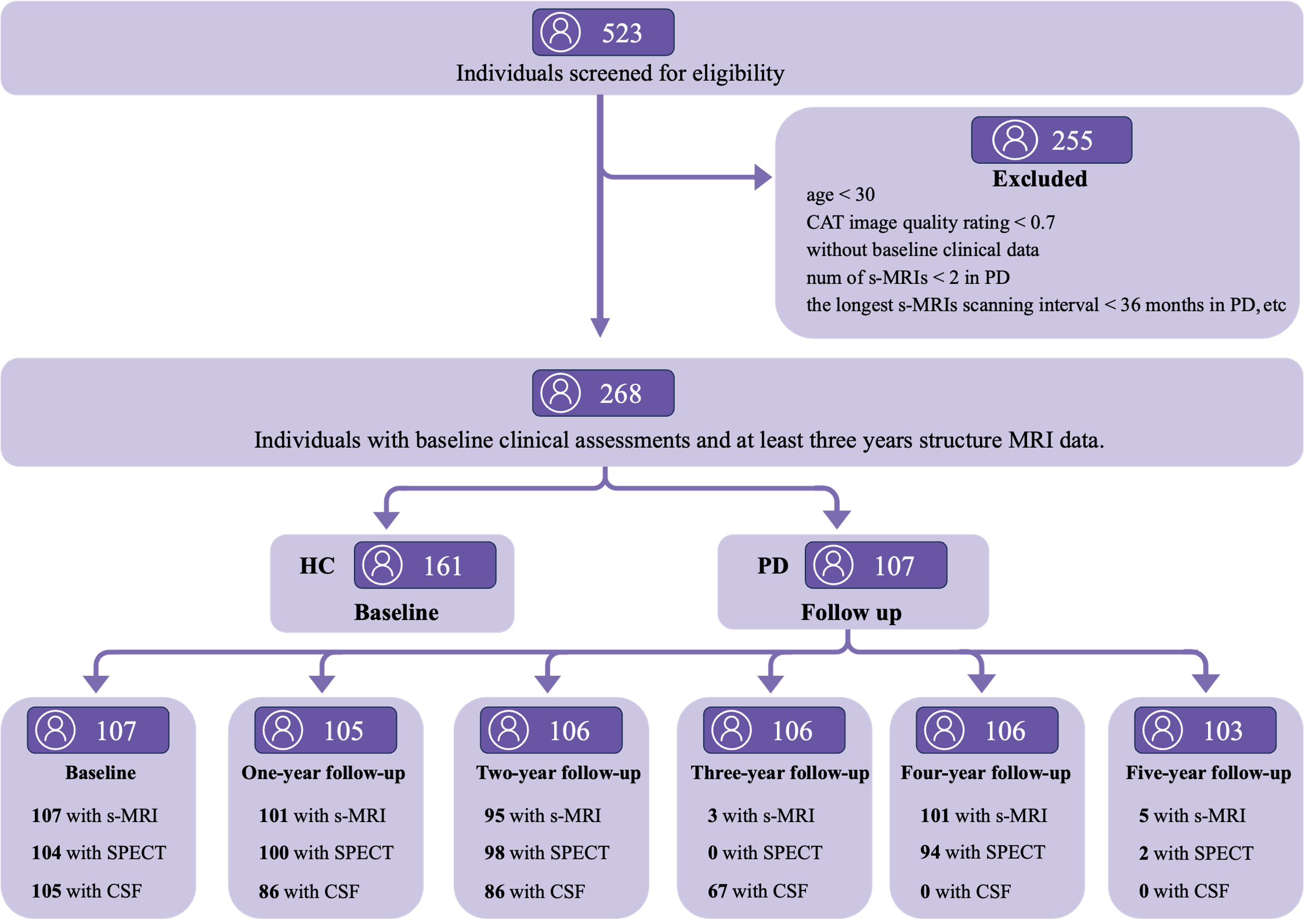


Supplementary Figure 1: Sample selection process and its overall information in five years of follow up. Participants were excluded if they withdrew from the study, failed imaging processing, failed image quality, the collected number of structural MRIs in patients fewer than two or the scanning interval less than 36 months. CAT Image Quality Rating below 70% didn't pass QC (quality control). CSF, cerebrospinal fluid; s-MRI, structural MRI; SPECT, single-photon emission computed tomography.


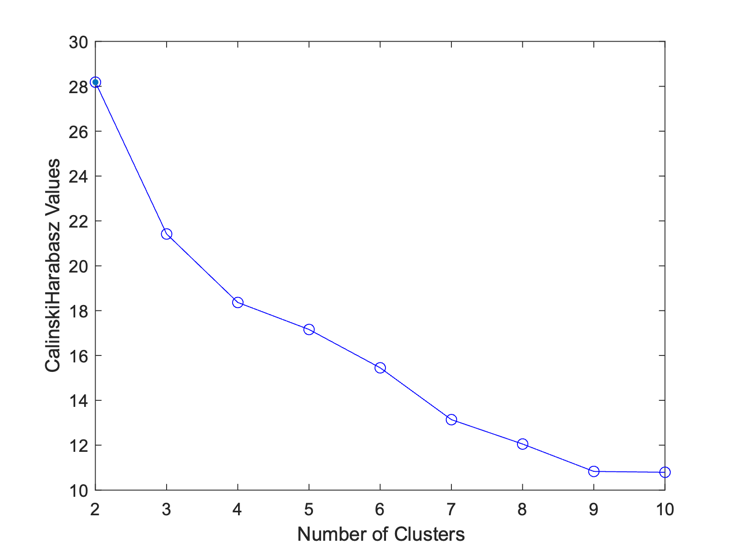


# Supplementary Figure 2: A graph representing the Calinski-Harabasz index for each potential number of clusters, suggesting that the optimal number of clusters is two.

**
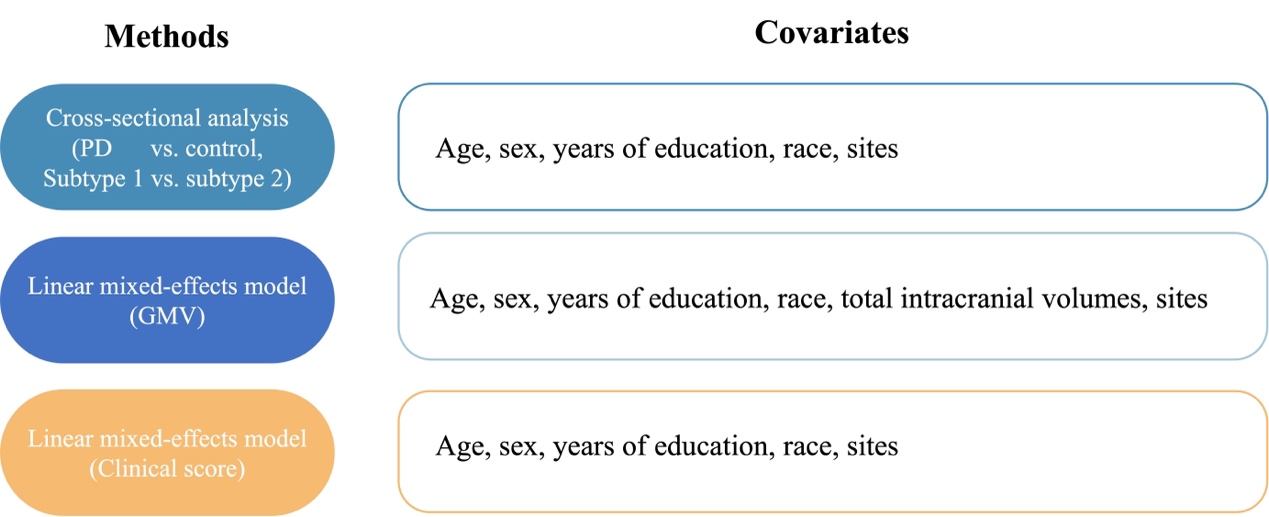
**

Supplementary Figure 3: Covariates utilized in the statistical analyses. Age, sex, years of education, race, site were adjusted in all analyses. In addition, for analysis involving neuroimaging data, total intracranial volumes were further added as covariates.

**
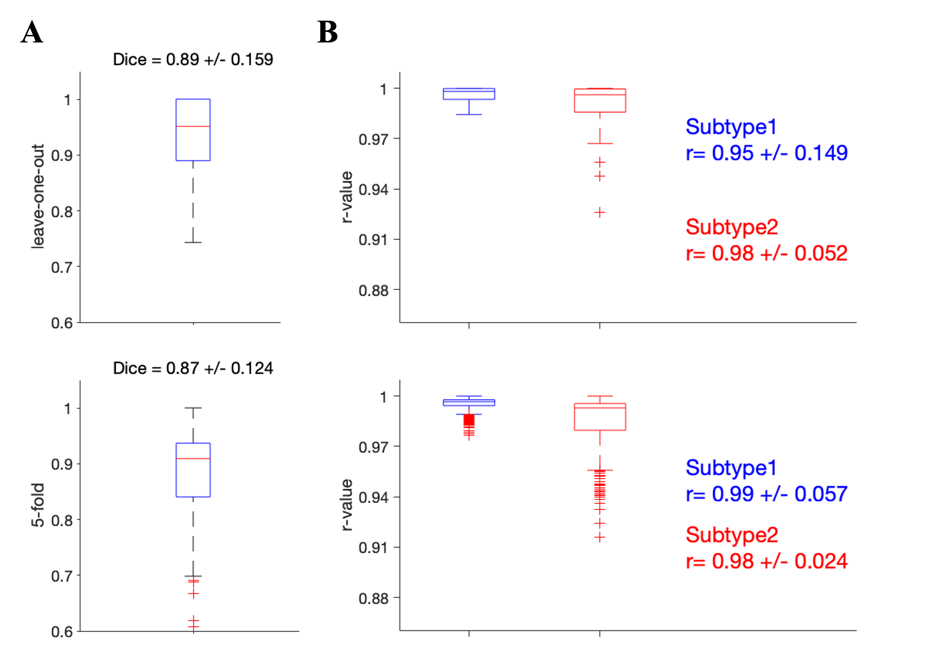
**

Supplementary Figure 4: Stability of cluster assignments and clustered atrophy rate-patterns. (A) Stability of cluster assignment. The upper panel exhibits the distribution of Dice's coefficients in the leave-one-out analysis. The lower panel shows the distribution of Dice's coefficients in the 5-fold cross-validation. (B) Stability of clustered atrophy rate patterns. The upper panel exhibits the distribution of the correlation coefficients in the leave-one-out analysis for each subtype. The lower panel shows the distribution of correlation coefficients in the 5-fold cross-validation for each subtype.


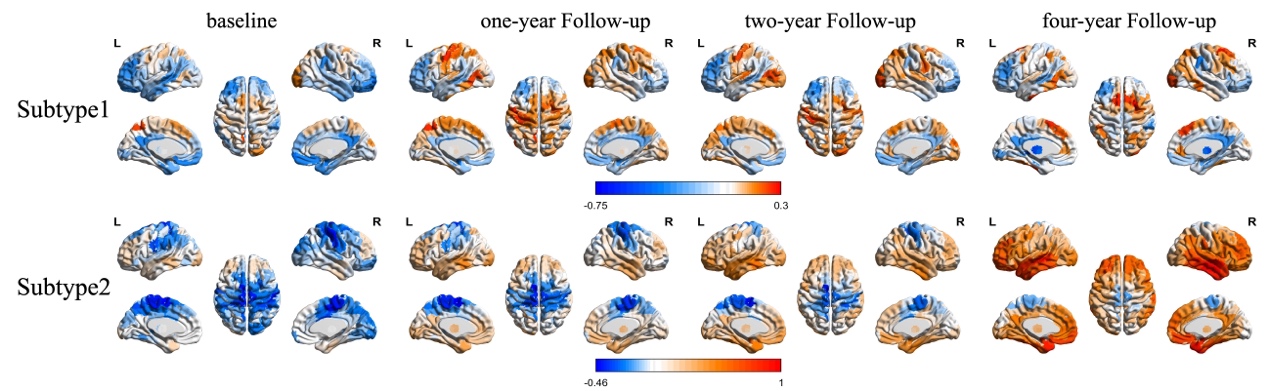


**Supplementary Figure 5: Comparison of atrophy patterns between two PD subtypes and healthy controls from baseline to four-year follow-up.** The GMV data were normalized by subtracting the mean GMV of the control group, resulting in higher z scores indicating more severe atrophy. The region-of-interest (ROI)-wise GMV values were adjusted by regressing out the effects of sex, age, race, total intracranial volume (TIV), and study site using a regression model.


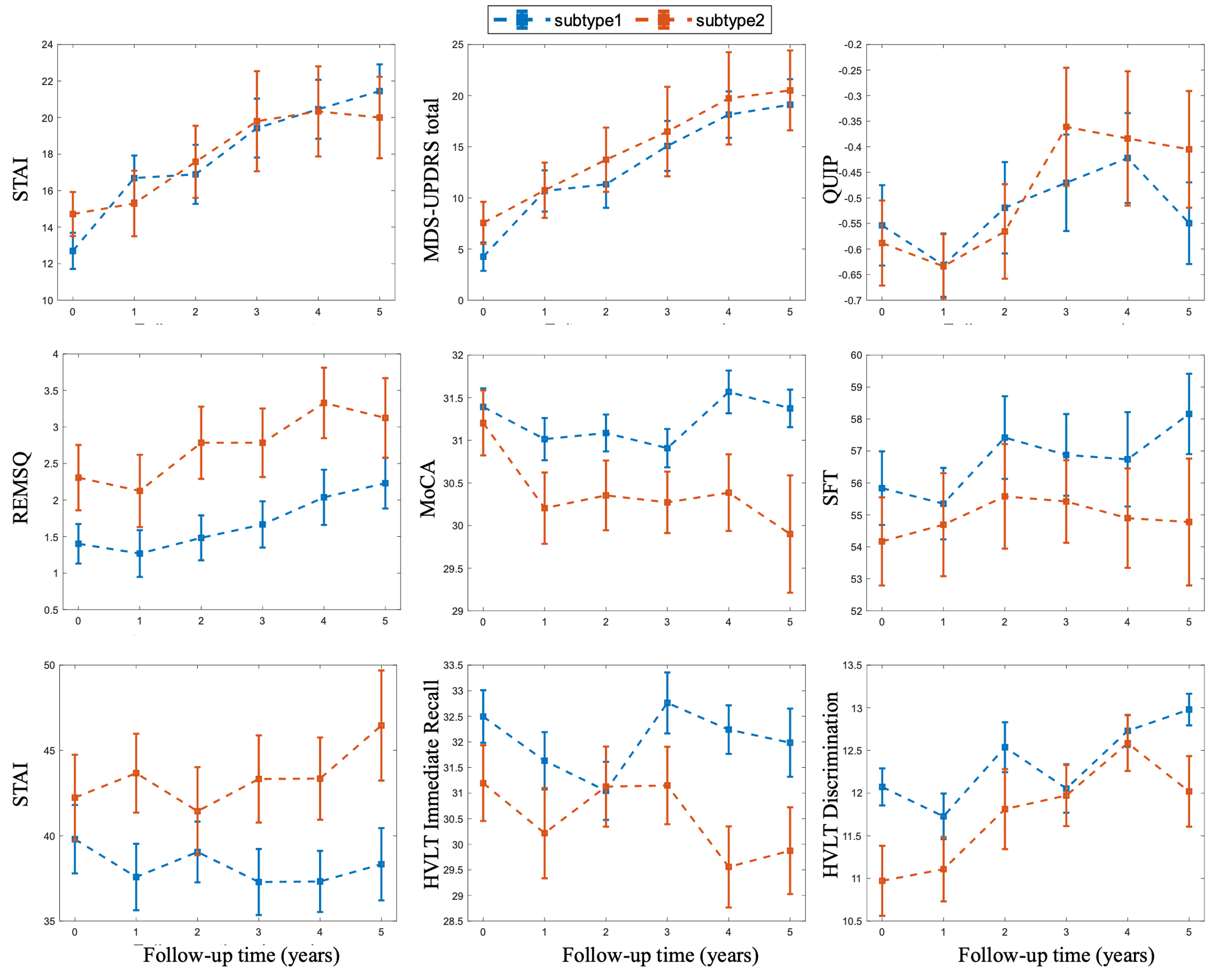


# Supplementary Figure 6: Longitudinal trajectories of clinical scores for two PD subtypes.

Asterisks indicate the statistical significance of the comparison between 2 subtypes in the clinical variables at the time of different follow up (*p < 0.05, **p< 0.01, ***p < 0.005, FDR correction in all follow-up time). HVLT = Hopkins Verbal Learning Test; MDS-UPDRS = Movement Disorder Society–sponsored revision of the Unified Parkinson’s Disease Rating Scale; MoCA = Montreal Cognitive Assessment; QUIP = Questionnaire for Impulsive-Compulsive Disorders in Parkinson’s Disease; RBD = REM sleep behavior disorder; RBDSQ = REM Sleep Behavior Disorder Screening Questionnaire; SCOPA-AUT = Scales for Outcomes in Parkinson’s Disease–Autonomic; STAI = State-Trait Anxiety Inventory.


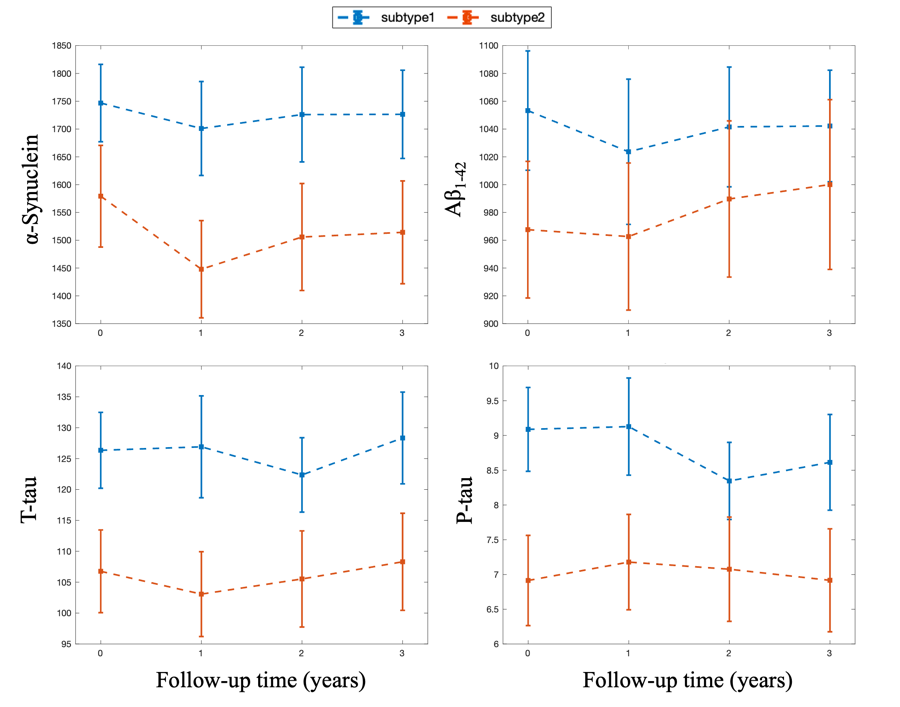


# Supplementary Figure 7: Longitudinal trajectories of CSF biomarkers for two PD subtypes.

Asterisks indicate the statistical significance of the comparison between 2 subtypes in the clinical variables at the time of different follow up (*p < 0.05, **p< 0.01, ***p < 0.005, FDR correction in all follow-up time), Aβ_1-42_ = β-amyloid 1-42; T-tau = total tau; P-tau = phosphorylated tau.
